# Assessment of the impact of reconstitution therapies—cladribine tablets and alemtuzumab—on the atrophy progression among patients with relapse-remitting multiple sclerosis

**DOI:** 10.3389/fnins.2025.1531163

**Published:** 2025-02-27

**Authors:** Aleksandra Pogoda-Wesołowska, Ignacy Stachura, Arkadiusz Zegadło, Marzena Maciągowska-Terela, Karolina Sobolewska, Aleksander Dębiec, Jacek Staszewski, Adam Stępień

**Affiliations:** ^1^Neurology Clinic, Military Institute of Medicine – National Research Institute, Warsaw, Poland; ^2^Faculty of Medicine, University of Warsaw, Warsaw, Poland; ^3^Department of Medical Radiology, Military Institute of Medicine – National Research Institute, Warsaw, Poland

**Keywords:** reconstitution therapies, multiple sclerosis, atrophy, volumetric changes, cladribine tablets, alemtuzumab

## Abstract

**Introduction:**

Immune reconstitution therapies (IRT) are highly effective therapies for multiple sclerosis (MS). Among IRT, we can distinguish partially selective therapies such as cladribine in tablets (CLAD) and non-selective therapies, which include alemtuzumab (ALEM). Today, it is known that these therapies are effective in controlling the relapse activity of the disease and the progression of clinical disability, which has been proven both in clinical trials and in real world evidence (RWE). However, there is a lack of data assessing the effect of IRT on the neurodegenerative process, which is intensified in patients with MS. The aim of the study was to assess the effect of IRT treatment on the degree and pattern of brain atrophy in patients with MS during 3 years of observation.

**Methods:**

Patients with relapsing-remitting MS (RRMS) treated with CLAD and ALEM were retrospectively recruited for the study. Demographic, clinical, and magnetic resonance imaging (MRI) data were collected at 4 time points: before the treatment and one, two, and three years after the treatment. MRI examinations were analyzed volumetrically using Freesurfer software. Global and regional changes in atrophy were assessed by calculating percentage changes in volume between time points. Results of drug groups were compared with each other.

**Results:**

After 3 years of follow-up, statistically significant differences between groups were observed in hippocampus [*p* < 0.01] and amygdala volume changes [*p* < 0.01]. Ventral diencephalon atrophy was noted in both groups. On the other hand, in both groups, no significant atrophy of white and grey matter was noted. In addition, an increase in the thalamus volume was observed.

**Discussion:**

In the studied groups, IRT therapies were shown to slow down the atrophy process in MS patients to a similar extent. These therapies may play a neuroprotective role by increasing the volume of the thalamus and hippocampus. The study was limited by the small number of both groups. Therefore, further studies are needed to fully assess the effect of reconstitution therapies on neurodegenerative processes in patients with RRMS.

## 1 Introduction

Multiple sclerosis (MS) is an inflammatory disease of the central nervous system (CNS) involving a complex combination of demyelination and neurodegeneration (Kotelnikova et al., 2017). MS has always been considered an inflammatory disease of the white matter (WM). Now, it is also known that gray matter (GM) involvement and the neurodegenerative mechanism are at least partially independent of inflammation. Despite significant progress in the treatment of patients with MS, the mechanisms causing the increase in disability are not fully understood. Over recent years, there has been remarkable progress in therapeutic approaches to inflammation. However, the ongoing neurodegenerative process is still little understood and undertreated. By assessing volume loss (atrophy) of individual brain areas, insight is gained into the potential role of these structures and the possibility of using them as biomarkers of disability progression in MS patients. The potential role of disease modifying therapies (DMTs) adds another level of complexity in the interpretation of factors leading to the progression of atrophy in MS (Sotirchos et al., 2020). Although such therapies are known to be effective in controlling relapsing disease activity and inhibiting the progression of clinical disability, data assessing their impact on the neurodegenerative process and its clinical expression in terms of cognition are largely lacking (Compston and Coles, 2002). Volumetric brain assessment is currently not widely used in clinical practice. However, it may become an important indicator in assessing the effectiveness of DMTs and the further course of the disease. Immune reconstitution therapies (IRT) are highly effective therapies for multiple sclerosis (MS). Among IRT, we can distinguish partially selective therapies such as cladribine in tablets (CLAD) and non-selective therapies, which include alemtuzumab (ALEM). Today, it is known that these therapies are effective in controlling the relapse activity of the disease and the progression of clinical disability, which has been proven both in clinical trials and in real world evidence (RWE) (Steingo et al., 2020; Ziemssen et al., 2020; Giovannoni et al., 2010; Costa-Frossard França et al., 2024; Stępień et al., 2023). However, there is a lack of data assessing the effect of IRT on the process of atrophy in patients with MS, and the reports available to date mainly refer to the assessment of the loss of the volume of the whole brain, without division into its individual areas (atrophy pattern) (Coles et al., 2017; Havrdova et al., 2017; Traboulsee et al., 2016; Filippi et al., 2000; De Stefano et al., 2018; Cortese et al., 2023). Currently, no analysis has been performed comparing the effect of CLAD and ALEM on the progression of brain volume loss and its atrophy pattern. The aim of the study was to assess the effect of IRT treatment on the degree and pattern of brain atrophy in patients with relapsing remitting MS (RRMS) during 3 years of observation.

## 2 Materials and methods

### 2.1 Participants

A retrospective, observational, longitudinal study was conducted at the Neurology Clinic of the Military Institute of Medicine – National Research Institute (MIM-NRI). Patients with relapsing–remitting multiple sclerosis (RRMS) diagnosed according to the 2017 McDonald criteria, treated with immune reconstitution therapies (IRT)—cladribine tablets (CLAD) and alemtuzumab (ALEM) were recruited to the study. Demographic data (age, gender, disease duration, comorbidities, number of previous therapies, adverse events, reason for treatment change) and clinical data (annualized relapse rate (ARR), Expanded Disability Status Scale (EDSS) score, number of new lesions on T2-weighted (T2) magnetic resonance imaging (MRI) including contrast-enhancing (Gd+) lesions) were collected, as well as MRI examinations at 4 time points: before treatment, 1 year after treatment, 2 years after treatment, and 3 years after treatment. Prior to statistical analysis, all the clinical, demographic and MRI data of patients were stripped of ID and PESEL number. Patients were marked with acronyms. This was a retrospective study and written consent from participants was not required. However, the project application was sent to the MIM-NRI Bioethics Committee. Resolution No. 54/24 (dated October 16, 2024) was issued, according to which the study was not subject to review by the Bioethics Committee.

### 2.2 MRI examination

All patients were scanned using the same MRI operating system on a General Electric Discovery MR750W3T 3.0 Tesla in the MIM-NRI Magnetic Resonance Imaging Laboratory. A standard protocol for MS was used, including sagittalis (sag) and axialis (ax) T2-weighted gradient-echo (T2) PROPELLER sequence, ax fluid-attenuated inversion recovery sequence (FLAIR), sag CUBE FLAIR sequence, ax diffusion-weighted imaging (DWI) sequence, three-dimensional (3D) susceptibility-weighted angiography (SWAN) sequence and ax 3D T1 pre- and post-contrast sequences. Baseline MRI examinations were performed before the initiation of therapy. As part of routine follow-up of new T2 lesions (including Gd+ lesions), yearly control MRI examinations were performed. MRI data were acquired from the hospital Alteris system as DICOM file folders in 0.6 mm axial 3D T1 and anonymized.

### 2.3 Brain segmentation

MRI examinations were analysed volumetrically using Freesurfer software (version 7.4.0).^1^ Segmentation of brain structures based on each subject 3D T1-weighted MRI was performed automatically for each patient using automated longitudinal FreeSurfer processing pipeline to obtain the cortical surface reconstruction and tissue-class segmentation boundaries. The original images were processed using the full longitudinal recon-all pipeline. This pipeline is designed to analyze repeated measures of the same patient. It uses a subject-specific template which reduces variability and increases statistical power, making it an appropriate tool to measure an individual’s atrophy. No manual editing was performed to keep methods as automated as possible, and scans with segmentation errors/failures were excluded. A total of 34 regions per hemisphere were segmented. The volume of the following anatomical regions: intracranial (excluding the volume of ventricles), the total, subcortical and cortical gray matter (GM), the total white matter (WM), cerebrum, cerebellum, brainstem, cerebrospinal fluid (CSF), ventricles along with different subcortical GM regions (thalamus, putamen, globus pallidus, accumbens, caudate nucleus, hippocampus, ventral diencephalon, amygdala) were extracted. For bilateral structures, the sums of the right and left volume fractions were used for analysis. For anatomical and subcortical tissue region labelling, a fully automated processing pipeline (FreeSurfer) was deployed (Fischl et al., 2002, 2004). FreeSurfer first affinely registered each T1-weighted MRI to a shared common space using MNI305 atlas (Collins et al., 1994). Next, the variation in the white matter intensity was quantified to remove the B1 bias field estimation. A skull stripped algorithm was then applied using a deformable template model (Ségonne et al., 2004). Following this nonlinear volumetric the MNI305 atlas, a simple label propagation algorithm was used to propagate the labels of the image template in the common atlas to the target-registered T1-weighted image (Fischl et al., 2002, 2004). The FreeSurfer-generated volumes were then measured following this step. Volumetric measurements were also manually verified by two qualified neurologists. MRI examinations performed after the age of 55 [due to the physiological annual loss of brain volume (approximately 0.2–0.4% per year)] and of poor quality (unable to analyze by Freesurfer software due to, for example, artefacts, different MRI protocol) were excluded from the analysis. Examinations performed after a switch to therapy different than CLAD or ALEM, and performed less than 8 weeks after intravenous steroid administration were also excluded. Assessment of the progression of brain atrophy was based on the comparison of volumes of different structures at different timepoints of the treatment period.

### 2.4 Statistical analysis

All volumes were obtained directly from the FreeSurfer output. For further analysis, volumes were normalized to the estimated total intracranial volume (which in the longitudinal FreeSurfer pipeline is the same for all scans corresponding to one patient). The values are therefore percentages of total intracranial volume, and as such are unitless. Then, for every structure, percentage change of volume (PCV) between corresponding time points was calculated as: (volume1 − volume2)/volume1 × 100%. To assess atrophy rates, the PCVs were analyzed. The normality of the data was tested using the Shapiro–Wilk test with an alpha level of 0.05. In most cases, the data were normally distributed and tested using two-sided one-sample *t*-tests against the null hypothesis of a zero mean. In non-normal cases, the two-sided Wilcoxon Signed-Rank test was used. For comparison of 3-year PCVs between two groups, two-sample *t*-tests were employed for normally distributed data, and the Mann–Whitney *U* test was applied for non-normal data. All hypothesis tests were conducted at a nominal alpha level of 0.05 per test. All statistical analyses were performed using the SciPy stats module.

Our study did not have a predefined hypothesis about an overall difference in atrophy rates between the two treatment groups across all brain structures. In other words, we did not aim to summarize the comparison between cladribine and alemtuzumab with a single overarching conclusion. Instead, the analysis focused on individual brain structures, treating each comparison as an independent analysis rather than controlling for the experiment-wise error rate (Bender and Lange, 2001). Given the absence of prior studies directly comparing the impact of cladribine and alemtuzumab on volume changes in multiple brain regions, we consider our results exploratory. Consequently, the reported *p*-values should be interpreted as descriptive and hypothesis-generating rather than confirmatory.

This was a pilot study retrospectively including data from all our patients. So far, studies on a similar population and with the use of the similar diagnostic methods have not been conducted. Therefore estimating the sample size *a priori* to demonstrate statistical significance would be burdened with a large error.

## 3 Results

Initially, 48 patients treated with CLAD and 19 patients treated with ALEM were recruited to the study. After excluding examinations performed in patients over 55 years of age, of low quality or poor sequence, performed in an inappropriate time interval, performed less than 8 weeks after intravenous steroid administration and in patients previously treated with natalizumab or ocrelizumab, data from 28 patients treated with CLAD and 19 patients treated with ALEM were included in the analysis. The initial comparison of groups is presented in Table 1. As can be seen, at baseline the treatment groups were similar in terms of age, duration of disease and number of previous disease modifying therapies (DMTs). In the CLAD group, most patients had previously been treated with dimethyl fumarate (58%), teriflunomide (12.9%) or fingolimod (9.6%), similarly to the ALEM group—dimethyl fumarate 33.3%, fingolimod 26.7% and interferon 2%. The CLAD group included one patient previously treated with natalizumab and one patient previously treated with ocrelizumab—they were excluded from the analysis. However, groups differed in terms of ARR and disability assessment on the EDSS scale—patients treated with ALEM were characterized by higher disease activity before the start of treatment.

**Table 1:** Baseline characteristics and comparison of treatment groups.

	CLAD (*n* = 28)	ALEM (*n* = 19)	*p*-value
Age (years, mean)	35.70	33.84	0.512
Duration of the disease (years, mean)	8.56	7.33	0.423
Number of previous DMTs (mean)	2.18	2.78	0.110
EDSS score	2.75	3.97	**0.004**
ARR	1.39	2.26	**0.008**
Number of new T2 lesions	3.75	6.26	**0.03**
Number of new Gd+ lesions	1.42	2.89	0.47

Statistically significant values are bolded. DMTs, disease modifying therapies; EDSS, Expanded Disability Status Scale; ARR, annualised relapse rate; T2, lesions–lesions on T2-weighted magnetic resonance imaging; Gd+, contrast-enhancing lesions.

In the CLAD group, the percentage of patients free from relapses increased from 13.3% before treatment to 76.7% after the first year of treatment, 81.8% after 2 years of treatment and was at the level of 72.3% after the third year of therapy. The mean number of new T2 and Gd+ lesions was reduced from 3.6 and 1.4 before treatment to 0.6 and 0.2 after the first year of CLAD therapy, to 0 after 2 years of treatment and was 0.9 and 0.2 after 3 years. The EDSS score, the median of which was 2.8 before treatment, increased to 3.0 after 1 year of therapy and remained stable after the second year of treatment, while after 3 years of therapy it was 3.5. After 3 years of treatment, 13.3% of patients achieved no evidence of disease activity (NEDA-3).

In the ALEM group, the percentage of patients free from relapses after 3 years of therapy was 75% and increased from 10.5% before treatment (after the first year it was 78.9% and after 2 years of treatment 94.4%). The mean number of new T2 and Gd+ lesions before treatment was 6.3 and 2.9 and was reduced to 0.3 and 0.1 after 1 year of therapy, to 0.2 and 0.1 after 2 years of treatment. After 3 years it was 0.9 and 0.5. The median EDSS score before treatment was 4.0 and decreased to 3.0 after 1 year of treatment, remaining stable after 2 and 3 years of treatment. After 3 years of therapy, NEDA-3 was observed in 31.6% of patients.

**Figure 1:**
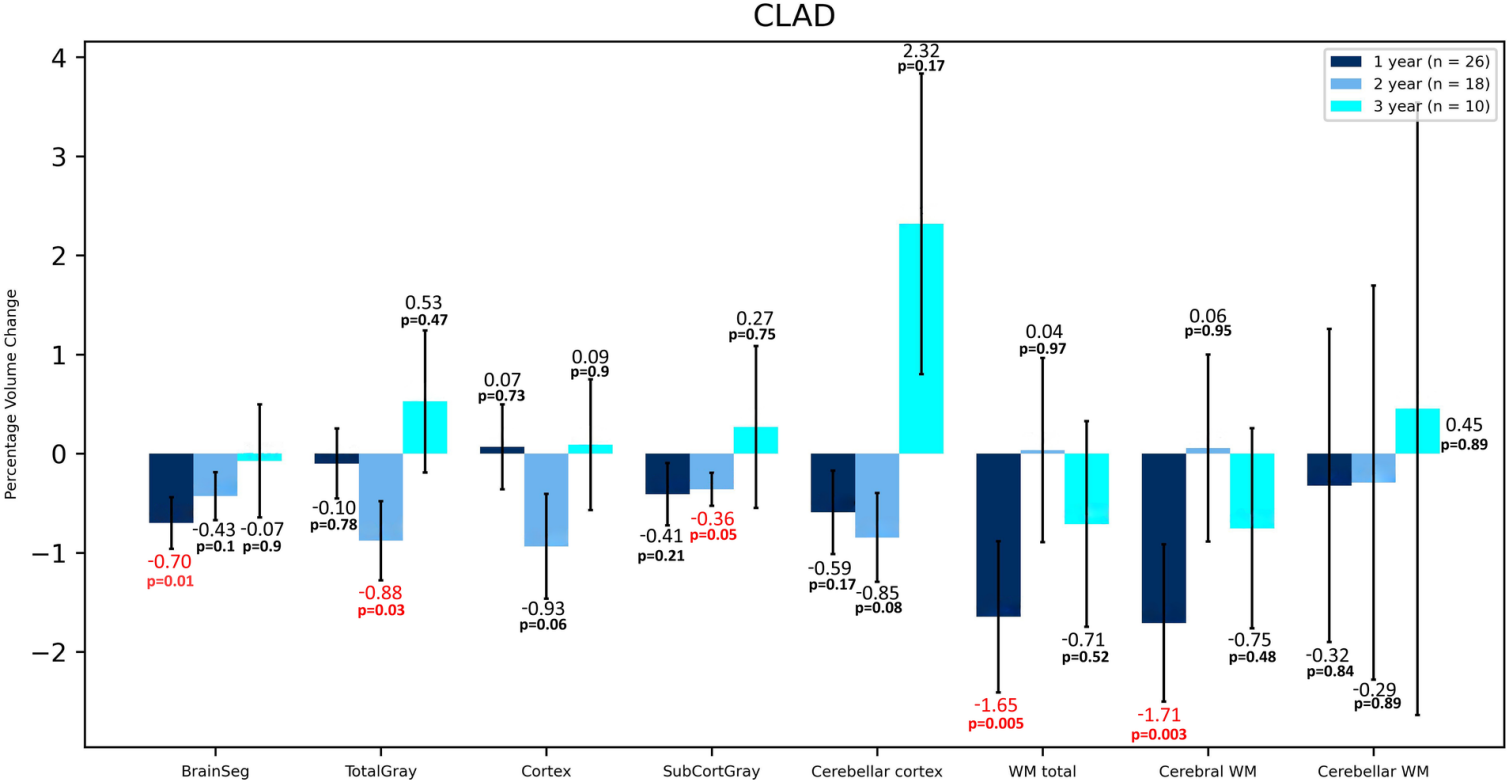
Volumetric changes during 3-years follow-up in patients treated with CLAD. BrainSeg, total brain volume; TotalGray, gray matter total volume; SubCortGray, subcortical gray matter volume; WM total, total volume of white matter; Cerebral WM, volume of white matter of cerebrum; Cerebellar WM, volume of white matter of cerebellum. Error bars represent standard error of the mean.

**Figure 2:**
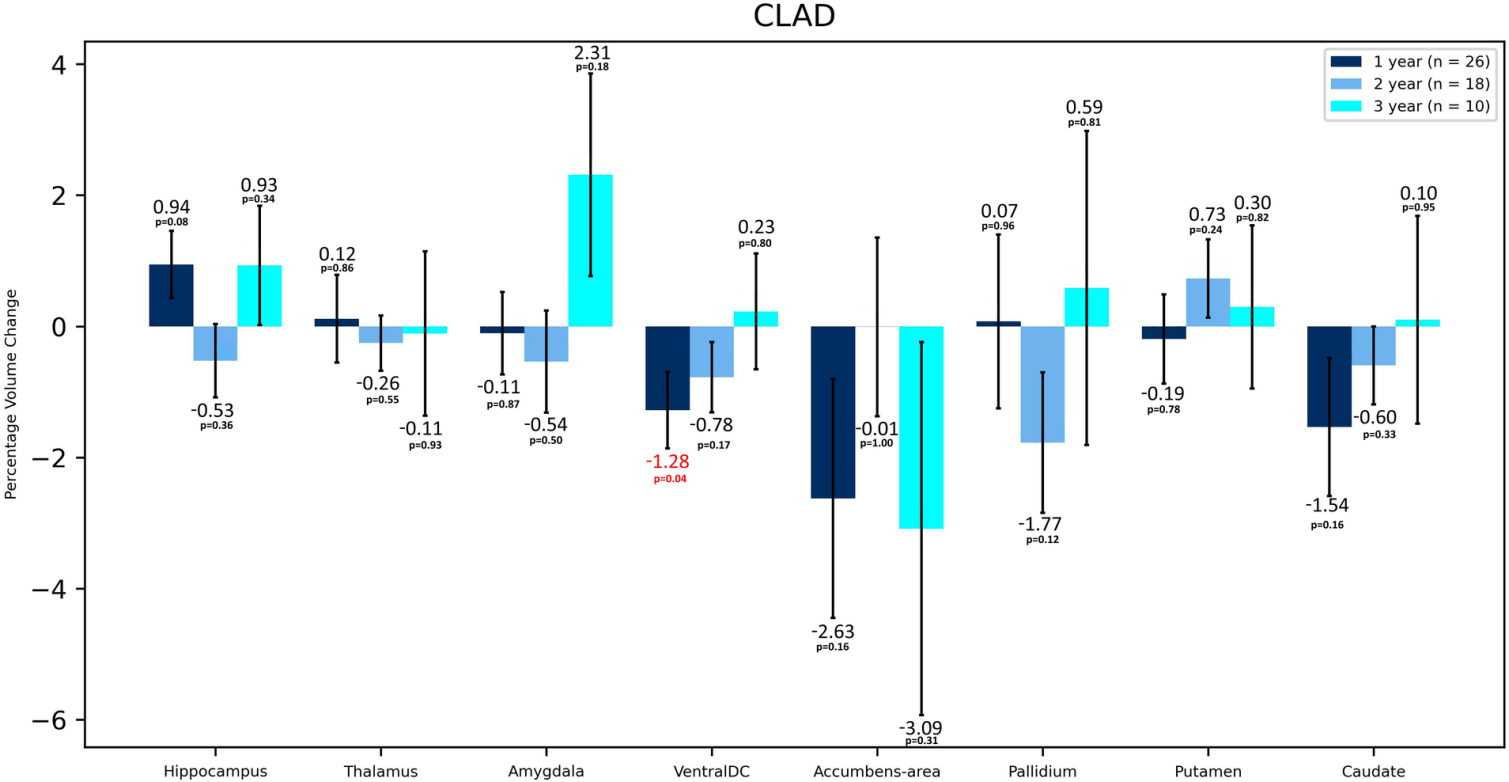
Volumetric changes during 3-years follow-up in patients treated with CLAD. Subcortical structures. VentralDC, ventral diencephalon. Error bars represent standard error of the mean.

In terms of volumetric analysis, a statistically significant atrophy of the total brain [−0.70%; *p* = 0.012] total WM [−1.65%; *p* = 0.005], including cerebral WM [−1.71%; *p* = 0.003] and ventral diencephalon (VDC) [−1.28%; *p* = 0.037] were noted in the CLAD group after the first year of therapy (Table 2 and Figure 1). A reduction in the loss of volume of these structures was observed in the subsequent years of treatment. The loss of total brain volume in the second year of treatment was reduced to −0.43%, and in the third year of therapy to −0.07%. After the second year of treatment a 0.04% increase in WM total volume was observed, whereas after 3 years of follow up its atrophy was −0.71%. As for cerebral WM atrophy, it also increased by 0.06% after the second year of treatment and after 3 years its atrophy was −0.75%. Inhibition of VDC atrophy was also noted—after 2 years of treatment it was −0.78%, and after the third year of follow-up its volume increased by 0.23%. No statistically significant atrophy of subcortical structures such as the nucleus accumbens, caudate nucleus, putamen, amygdala or corpus callosum was noted, and even a reduction of their atrophy or an increase in volume in the following years was observed (Table 2 and Figure 2). After 3 years of treatment, no significant atrophy of GM, subcortical GM and cortex was noted, and even an increase in their volumes the third year of therapy were observed. In the three-year follow-up, statistically significant atrophy of the VDC [−2.98%; *p* = 0.017] was noted (Table 2). Interestingly, an increase in the volume of the hippocampus by 2.37% was observed, which was statistically significant [*p* = 0.005]. In addition, an increase in the thalamus [by 0.28%], amygdala [by 0.02%], pallidium [by 0.39%] and corpus callosum [by 8.22%] volumes were also noted.

**Table 2:** Annual volumetric changes in the CLAD group.

	3 year period (*n* = 8)			1 year (*n* = 28)			2 year (*n* = 17)			3 year (*n* = 8)		
	Mean	*p* value	Std	Mean	*p* value	Std	Mean	*p* value	Std	Mean	*p* value	Std
BrainSeg	-1.132	0.149	1.978	-0.699	0.012	1.378	-0.428	0.096	0.998	-0.073	0.901	1.611
TotalGray	−2.192	0.095	3.216	−0.100	0.779	1.863	−0.879	0.031	1.648	0.527	0.486	2.026
Cortex	−2.492	0.120	3.976	0.069	0.728	2.262	−0.935	0.057	2.175	0.090	0.895	1.866
SubCortGray	−0.863	0.294	2.154	−0.408	0.206	1.667	−0.360	0.046	0.687	0.270	0.751	2.310
Cerebellar cortex	−1.477	0.418	4.857	−0.592	0.171	2.226	−0.846	0.078	1.850	2.318	0.170	4.289
WM total	0.153	0.547	6.354	−1.646	0.005	4.035	0.035	0.970	3.829	−0.710	0.516	2.934
Cerebral WM	0.371	0.547	6.639	−1.708	0.003	4.195	0.056	0.953	3.890	−0.753	0.480	2.854
Cerebellar WM	−3.836	0.050	4.593	−0.322	0.840	8.353	−0.292	0.885	8.197	0.453	0.888	8.740
WM-hypo	−9.371	0.163	17.019	−4.197	0.268	19.655	9.703	0.027	22.650	8.251	0.742	28.164
Hippocampus	2.366	0.005	1.637	0.945	0.077	2.719	−0.525	0.361	2.303	0.930	0.341	2.573
Thalamus	0.277	0.896	5.770	0.116	0.863	3.538	−0.256	0.552	1.734	−0.109	0.933	3.550
Amygdala	0.022	0.990	4.682	−0.105	0.868	3.322	−0.540	0.498	3.212	2.312	0.178	4.370
VentralDC	−2.980	0.017	2.716	−1.280	0.037	3.085	−0.777	0.167	2.215	0.227	0.805	2.500
Accumbens-area	−6.264	0.109	9.638	−2.625	0.161	9.638	−0.008	0.995	5.622	−3.086	0.314	8.048
Pallidium	0.392	0.932	12.521	0.075	0.955	7.008	−1.774	0.118	4.428	0.586	0.814	6.778
Putamen	−0.739	0.763	6.655	−0.194	0.778	3.611	0.730	0.240	2.464	0.297	0.818	3.520
Caudate	−2.897	0.231	6.249	−1.536	0.157	5.577	−0.596	0.332	2.454	0.099	0.952	4.482
CC	8.216	0.742	28.027	−0.932	0.652	10.834	0.599	0.747	3.769	4.350	0.353	12.367
Lateral ventricle	12.643	0.081	17.576	4.177	0.056	10.057	1.943	0.124	4.939	3.587	0.360	10.368
VentricleChoroidVol	11.106	0.101	16.662	3.799	0.099	9.893	1.912	0.040	4.918	3.098	0.412	10.051
CSF	−2.492	0.534	10.772	−0.559	0.451	8.656	1.446	0.351	6.206	−2.194	0.063	2.815

Statistically significant changes are marked in green.

**Figure 3:**
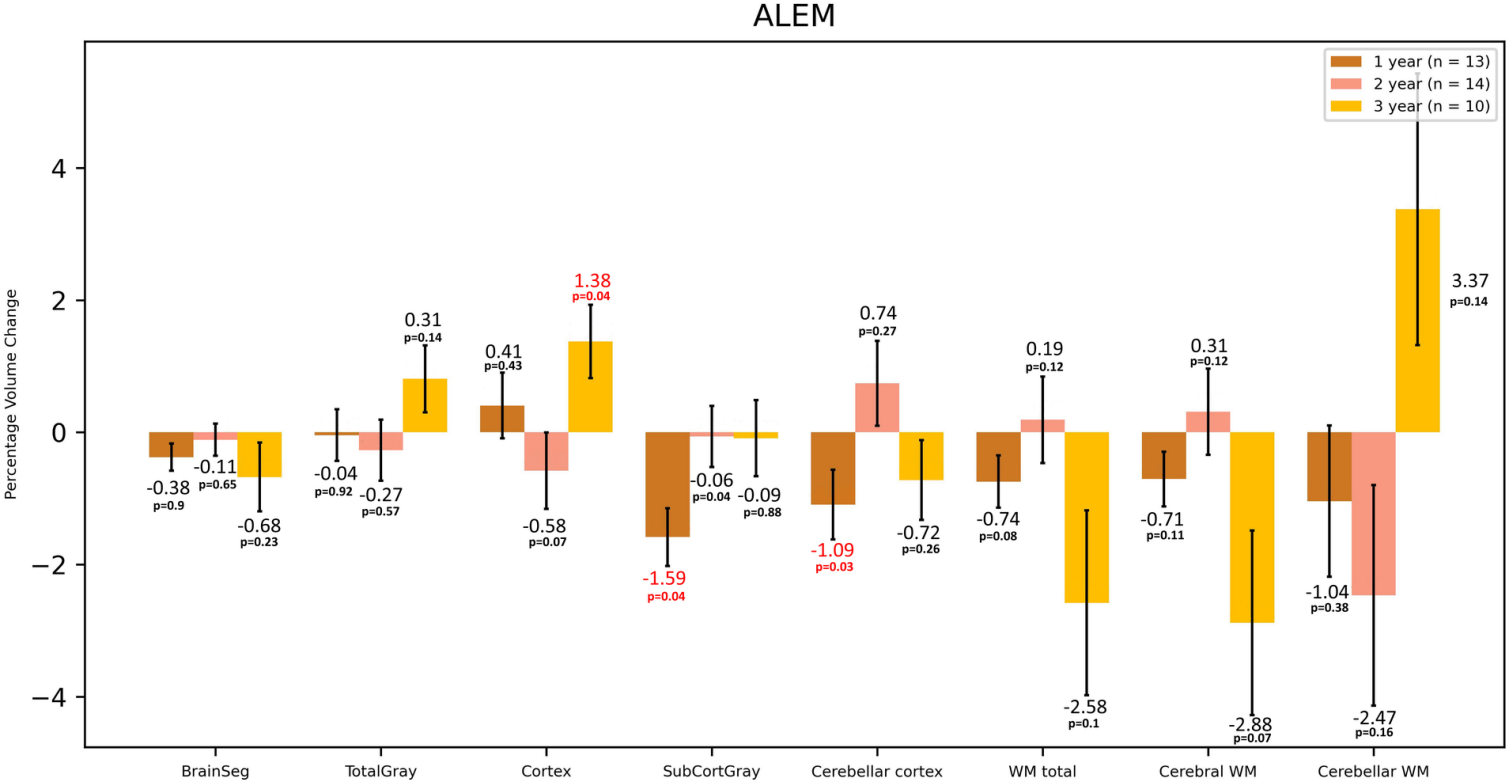
Volumetric changes during 3-year follow-up in patients treated with ALEM. BrainSeg, total brain volume; TotalGray, gray matter total volume; SubCortGray, subcortical gray matter volume; WM total, total volume of white matter; Cerebral WM, volume of white matter of cerebrum; Cerebellar WM, volume of white matter of cerebellum. Error bars represent standard error of the mean.

**Figure 4:**
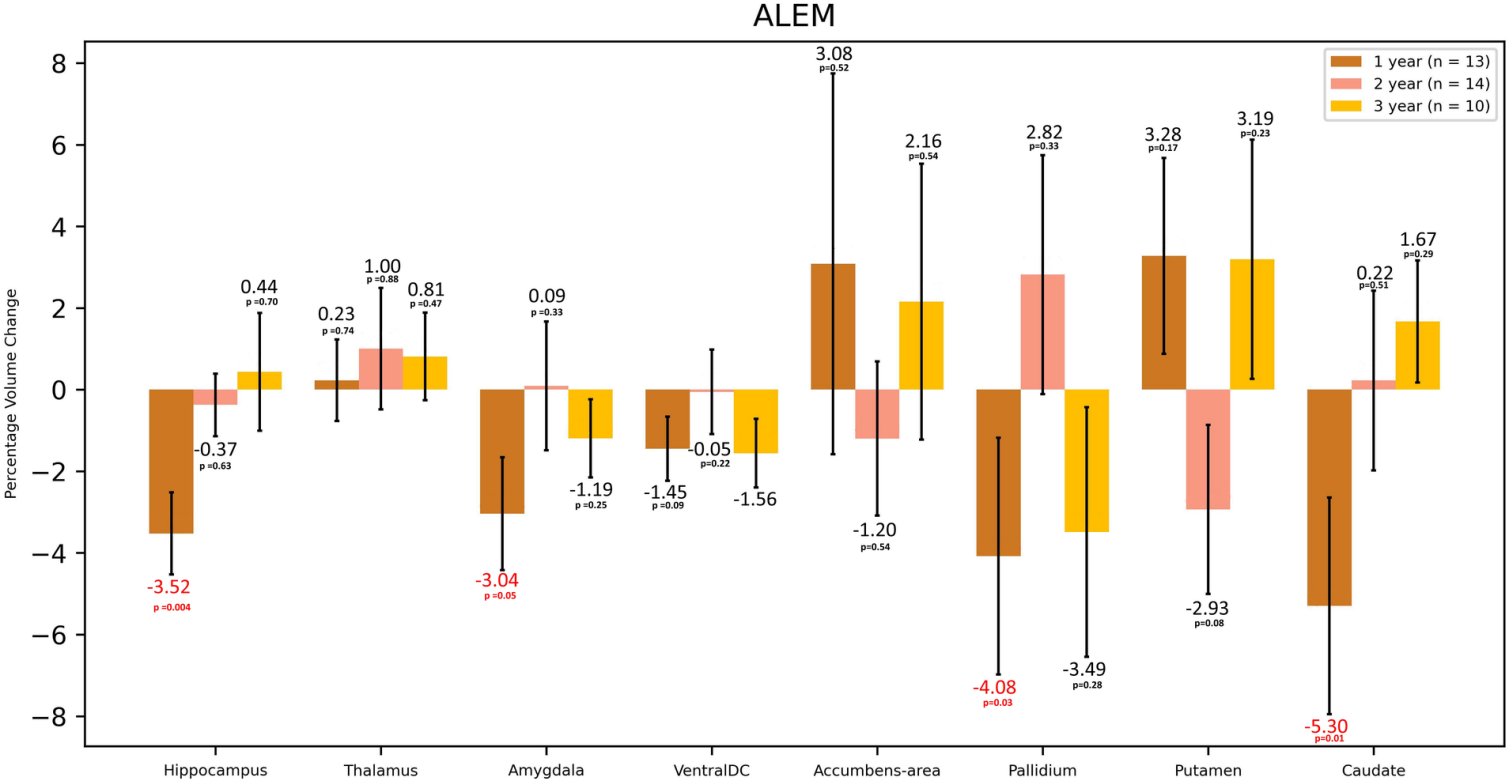
Volumetric changes during 3-years follow-up in patients treated with ALEM. Subcortical structures. VentralDC, ventral diencephalon. Error bars represent standard error of the mean.

In the case of the ALEM-treated group, the greatest WM atrophy was observed only after 3 years of therapy [−2.58%], but it was not statistically significant [*p* = 0.10] (Table 3 and Figure 3). Interestingly, after the third year of follow-up, a statistically significant reduction in the volume of hypointense WM lesions (WM-hypo) was also noted (− 22.97%), which was statistically significant [*p* = 0.012]. In the first year of treatment, statistically significant atrophy was observed in the amygdala [−3.04%; *p* = 0.049], pallidum [−4.08%; *p* = 0.027], hippocampus [−3.52%; *p* = 0.004], caudate nucleus [−5.30%; *p* = 0.013], as well as subcortical GM [−1.59%; *p* = 0.003] and cerebellar cortex [−1.10%; *p* = 0.033] (Table 3 and Figure 4). This atrophy slowed down and even an increase in the volume of some of these structures was observed in the subsequent years of treatment. Importantly, in the subsequent years of treatment, a slowdown in cortical and subcortical GM atrophy was observed, and even a statistically significant increase in the cortex volume was noted in the third year of observation [by 1.38%; *p* = 0.035]. In the three-year follow-up, statistically significant atrophy was observed in the total brain volume [1.16%; *p* = 0.049], amygdala [−7.93%; *p* = 0.008], WM-hypo [−21.57%; *p* = 0.016] and VDC [−3.30%; *p* = 0.010] (Table 3). Interestingly, an increase in the volume of total GM [by 0.26%] and thalamus [by 1.19%] after 3 years of treatment was noted. The same applied to the accumbens area [volume increase by 5.38%], and putamen [volume increase by 0.17%].

**Table 3:** Annual volumetric changes in the ALEM group.

	3 year period (*n* = 7)			1 year (*n* = 13)			2 year (*n* = 14)			3 year (*n* = 10)		
	Mean	*p* value	Std	Mean	*p* value	Std	Mean	*p* value	Std	Mean	*p* value	Std
BrainSeg	−1.164	0.049	1.253	−0.376	0.093	0.742	−0.111	0.651	0.900	−0.675	0.227	1.647
TotalGray	0.259	0.833	3.114	−0.042	0.916	1.406	−0.272	0.566	1.727	0.809	0.144	1.599
Cortex	1.279	0.452	4.209	0.405	0.430	1.791	−0.581	0.074	2.162	1.376	0.035	1.756
SubCortGray	−2.462	0.105	3.415	−1.585	0.003	1.576	−0.064	0.510	1.724	−0.090	0.880	1.819
Cerebellar cortex	−2.167	0.217	4.154	−1.095	0.033	1.902	0.739	0.271	2.404	−0.723	0.262	1.908
WM total	−2.988	0.208	5.597	−0.745	0.083	1.420	0.190	0.124	2.448	−2.581	0.098	4.421
Cerebral WM	−3.069	0.215	5.852	−0.706	0.113	1.488	0.311	0.124	2.433	−2.881	0.069	4.417
Cerebellar WM	−1.074	0.589	4.989	−1.040	0.382	4.129	−2.466	0.163	6.240	3.374	0.135	6.494
WM-hypo	−21.566	0.016	22.796	3.848	0.455	20.104	−0.246	0.950	14.376	−22.972	0.012	23.322
Hippocampus	−3.572	0.101	4.887	−3.525	0.004	3.627	−0.372	0.634	2.862	0.439	0.695	4.567
Thalamus	1.192	0.702	7.869	0.231	0.735	3.597	1.001	0.875	5.567	0.813	0.468	3.395
Amygdala	−7.926	0.008	5.345	−3.041	0.049	4.994	0.092	0.331	5.911	−1.193	0.245	3.034
VentralDC	−3.303	0.010	2.360	−1.447	0.091	2.842	−0.054	0.221	3.867	−1.557	0.097	2.654
Accumbens-area	5.376	0.578	14.602	3.084	0.521	16.817	−1.197	0.537	7.063	2.157	0.539	10.693
Pallidium	−4.770	0.404	14.058	−4.078	0.027	10.468	2.818	0.331	10.945	−3.485	0.284	9.666
Putamen	0.172	0.961	8.829	3.276	0.168	8.656	−2.935	0.074	7.745	3.193	0.232	9.270
Caudate	−0.937	0.375	11.167	−5.298	0.013	9.567	0.224	0.510	8.234	1.669	0.294	4.732
CC	5.517	0.642	29.867	5.689	0.340	26.378	4.072	0.730	22.834	−1.240	0.592	7.061
Lateral ventricle	3.588	0.715	24.781	3.442	0.103	7.025	−0.015	0.245	12.254	1.322	0.492	12.160
VentricleChoroidVol	3.682	0.713	25.263	4.243	0.032	6.318	0.354	0.177	12.164	1.243	0.432	12.734
CSF	−11.271	0.109	15.828	−1.078	0.713	10.335	1.733	0.087	17.304	−3.072	0.478	13.124

Statistically significant changes are marked in green.

**Figure 5:**
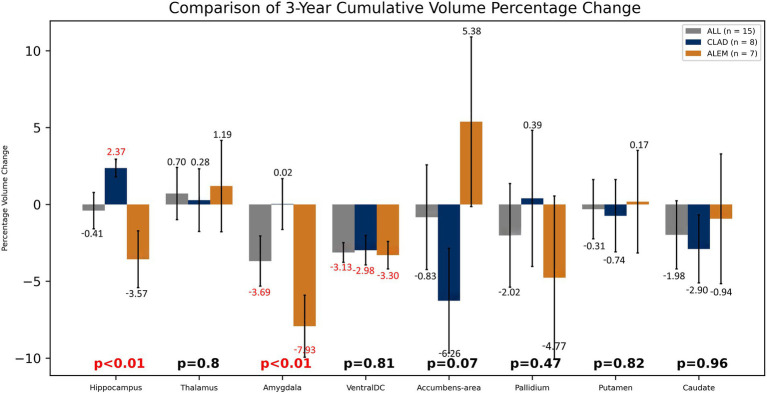
Comparison of both drug groups in 3-year follow-up. Error bars represent standard error of the mean.

**Figure 6:**
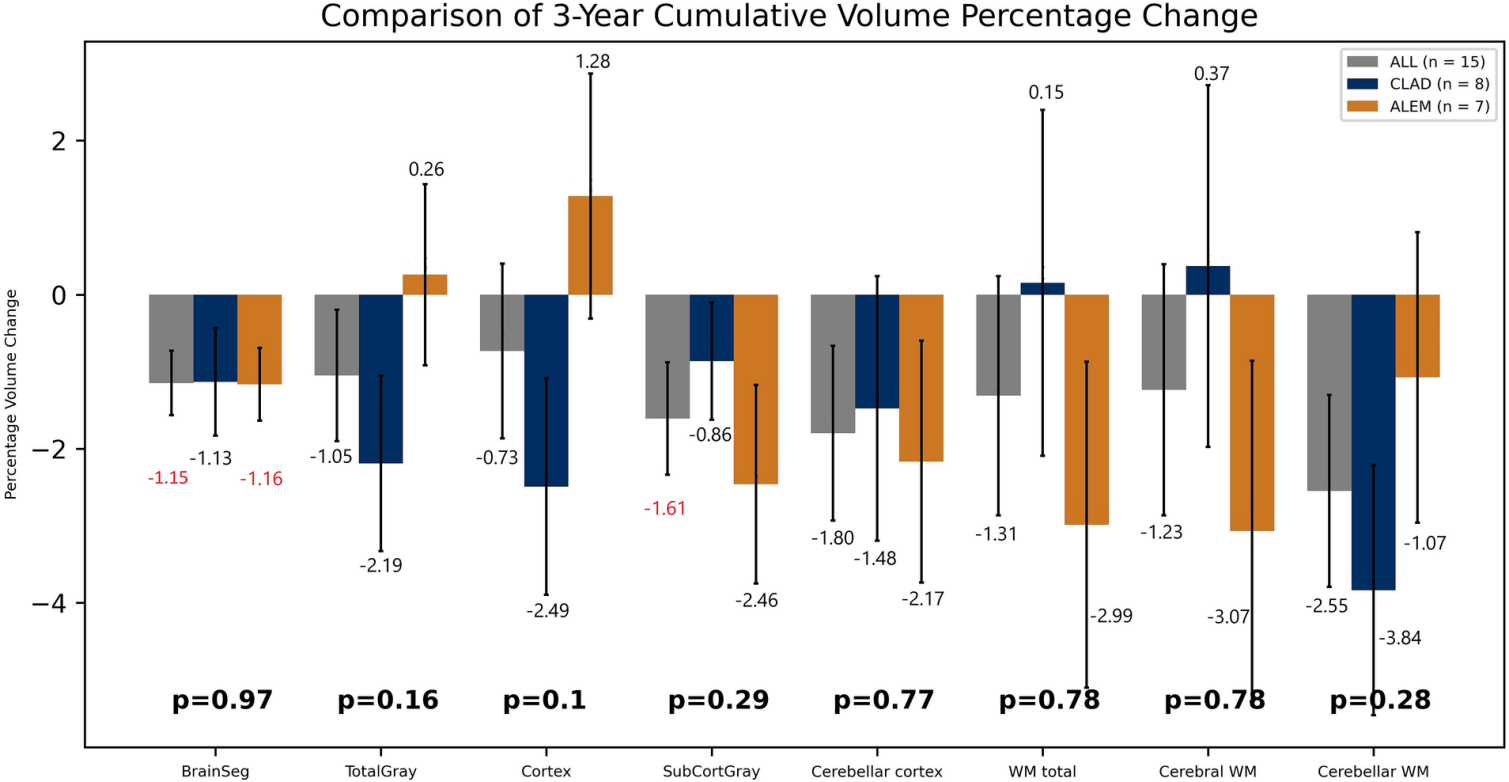
Comparison of both drug groups in 3-year follow-up. BrainSeg, total brain volume; TotalGray, gray matter total volume; SubCortGray, subcortical gray matter volume; WM total, white matter total volume; Cerebral WM, volume of cerebrum white matter; Cerebellar WM, volume of cerebellum white matter. Error bars represent standard error of the mean.

In the comparison of both groups after 3 years of observation, statistically significant differences were observed in the hippocampus volume changes—an increase of 2.37% in the CLAD group and a decrease of 3.57% in the ALEM group [*p* < 0.01] and amygdala volume changes– a increase by 0.02% in the CLAD group and 7.93% in the ALEM group [*p* < 0.01] (Figure 5) Interestingly, VDC atrophy was observed in both groups. However, what is noteworthy, the clear increase in the volume of the cortex [by 1.28%] and GM [by 0.26%] volumes in the ALEM-treated group was observed compared to the CLAD group, where a decrease in the volume of these structures [respectively −2.49% and −2.19%] was noted during the three-year follow-up. Fortunately, significant atrophy of GM, WM and cortex throughout all years of observation was not noted in both groups (Figure 6). Moreover, in both groups, an increase in the volume of the thalamus was reported (Figure 5). The results of the group comparison for all analyzed brain structures, along with the corresponding Cohen’s d coefficients and post-hoc power analysis, are provided in Supplementary Table S1.

However, after excluding the first year of observation from the analysis, the only statistically significant difference concerned the changes in the volume of WM-hypo lesions—in the CLAD group an increase by 12.10%, in the ALEM group a reduction by 17.35% [*p* = 0.002]. Interestingly, the greatest reduction in WM-hypo lesions in the ALEM-treated group was noted in the third year of follow-up [−22.97%], which was associated with the greatest reduction in WM volume during this period [−2.58]. Conversely, in the CLAD-treated group, an increase in the volume of WM-hypo lesions [by 8.25%] was observed in the third year of follow-up, which was associated with the inhibition of WM atrophy after 3 years of follow-up in this group.

Disease duration, patient’s age, and the number of prior therapies are crucial factors to consider in larger datasets. However, due to the limited sample size (15 patients over a 3-year period), conducting a comprehensive multivariate analysis to assess the influence of these factors in this research was not feasible without compromising statistical validity. Primary focus in this analysis was to compare the effects of CLAD and ALEM on brain atrophy progression. Although these additional factors were not included directly in the statistical analysis, key characteristics were compared at baseline. Importantly, they were well-matched between the two groups, reducing the risk of confounding effects. Nevertheless, an additional analysis to explore the potential impact of these factors was performed. Using a generalized linear model (GLM), we investigated how age, the number of previous therapies, and treatment group (cladribine or alemtuzumab) explained brain atrophy observed during the first year. This analysis was limited to the first year to obtain approximately 15 observations per factor. It was conducted separately for all brain structures that showed a statistically significant volume change during the first year in either treatment group. In this additional analysis, age was a statistically significant factor for subcortical GM volume changes (*p* = 0.024), while the treatment group significantly affected hippocampus volume changes (*p* = 0.001). For all other structures, none of the factors reached statistical significance. The additional GLM analysis supported our primary findings by confirming that, for structures like the hippocampus and amygdala—where significant differences were observed—the treatment effect was the dominant factor explaining the differences between the two groups.

In the CLAD group, the NEDA-4 index was noted in 13.3% of patients and in the ALEM group in 15.8% of patients after 3 years of follow-up.

## 4 Discussion

The results of our study indicate that both CLAD and ALEM may contribute to inhibiting the atrophy process in patients with RRMS. The obtained results also suggest that these therapies may demonstrate a neuroprotective character by increasing the volume of structures such as the hippocampus or thalamus. The mechanism of this action is not entirely clear. There is no available literature explaining this process. However, these observations are consistent with the study of Rekik et al. (2022) where the authors observed an increase in the volume of the thalamus. Also in the study of Raji and Winkler (2023), an increase in the volume of WM, thalamus, corpus callosum and hippocampus, in patients who were treated with CLAD, was observed. It is possible that the process of remyelination may also have an impact on this, which was described in a research reporting the effect of dimethyl fumarate on oligodendrocytes *in vitro* (Guerriero et al., 2022). Interestingly, in the Dos Passos study the myelin content changes in lesions of patients with highly active RRMS was retrospectively assessed at baseline and after 2 years of CLAD treatment using the MRI-based q-Space5 myelin map (Dos Passos et al., 2024). Remyelination occurred in 27.1% of lesions in participants who achieved NEDA-3, compared with 12.0% in others (*p* < 0.001).

Furthermore, in both drug groups, no significant atrophy of the GM and cortex was noted, which may indicate their effect on saving its volume. Moreover, in the ALEM-treated group, an increase in the volume of GM and cortex was noted after 3 years of therapy, which in the case of cortex was statistically significant in the third year of observation. Several histopathological and MRI studies have shown that significant global cortical thinning is a diffuse and early phenomenon in MS (Azevedo et al., 2015). This correlates with clinical disability and is partially independent of pathological inflammation in WM. Moreover, both CLAD and ALEM groups showed an increase in thalamic volume at 3 years of follow-up. According to previous studies, of all subcortical GM areas, the thalamus is the most susceptible to atrophy. Loss of thalamic tissue volume has been found in all subtypes of MS (Chataway, 2010). This may prove the slowing down of atrophy and the neuroprotective nature of IRT.

However, during the three-year follow-up, statistically significant atrophy of the VDC was observed in both groups. VDC is not an anatomical name for a single structure, but rather a name for a group of structures that usually cannot be distinguished from each other using standard MRI images (Caviness et al., 1999). This area includes the hypothalamus, mammillary body, subthalamic nuclei, substantia nigra, red nucleus, lateral geniculate nucleus (LGN), and medial geniculate nucleus (MGN). White matter areas such as the zona incerta, cerebral peduncle (crus cerebri), lenticular fasciculus, and the medial lemniscus are also included in this area. The optic tract is included in this area to the most anterior extent. Each structure fades in and out of the VDC at different times. Therefore, the VDC greatly varies from slice to slice. This may therefore cause large errors in measuring this area and, consequently, difficulties in interpreting changes in its volume.

According to the available literature, increased atrophy in the first year of CLAD treatment, which was not observed in the subsequent years of observation, may be associated with the phenomenon of pseudoatrophy (Cortese et al., 2023). In the study by Cortese et al. (2023), after a short period of atrophy (6 months), GM volume loss progressed at a slower rate in RRMS patients treated with CLAD than in those treated with placebo. The authors explained the atrophy occurring up to 6 months of follow-up as a pseudoatrophy phenomenon—patients treated with anti-inflammatory drugs may experience a rapid decrease in brain volume in the first months of treatment due to the resolution of inflammatory edema and probably also changes in the volume of inflammatory cells and soluble neurotoxic factors. The time course of pseudoatrophy is not fully understood and it can occur for up to 1 year in some conditions (Sastre-Garriga et al., 2020). The presence of ongoing inflammatory activity in MS leads to fluctuations in the entire brain volume, and mainly in WM volume, due to both fluid and inflammatory infiltrates. Paradoxically, attenuation of this inflammation by DMTs causes marked atrophy in the first period of treatment (so-called “pseudoatrophy”). In the long term, inflammation may lead to greater demyelination and neuroaxonal degeneration, leading to greater brain tissue loss. This suggests that the neuroprotective effects of most DMTs may be largely secondary to their anti-inflammatory effects with a delayed reduction in the rate of atrophy, which should be taken into account when assessing the potential neuroprotective effects of these therapies (Koudriavtseva and Mainero, 2016). The presented results confirm this phenomenon indicating that after exclusion of the first year of observation from the analysis the only statistically significant difference concerned the changes in the volume of WM-hypo lesions. Moreover, in most structures atrophy was significant only in the first year of treatment and was not noted in the subsequent years of the follow-up. Furthermore, in the ALEM-treated group, the biggest WM atrophy was noted after 3 years of follow-up, which was associated with significant atrophy of WM-hypo lesions and may be related to the anti-inflammatory effect. According to current reports, the volumes of white matter hyperintense lesions on FLAIR sequence (WM-hyper) and WM-hypo lesions in T1 sequence in MRI examination may be similar (Wei et al., 2019). Freesurfer software includes WM-hypo lesions to WM total volume. The analysis by Wei et al. (2019) confirmed that WM-hypo derived from T1 images acquired for 3D volumetric analysis is essentially equivalent to WM-hyper derived from T2-FLAIR. FreeSurfer-generated WM-hypo volumes, which are rarely used today, may serve as a reliable measure of WM damage. Perhaps, the most visible decrease in WM total volume in the third year of follow-up in the ALEM-treated group is associated with a strong reduction in WM-hypo lesions, which would indicate the development of a strong anti-inflammatory effect of ALEM.

To the best of our knowledge, the comparative analysis presented here is the first study of this type comparing the effect of two different IRTs—CLAD and ALEM—on the degree of atrophy in patients with RRMS. To date, there are also no reports assessing so detailed the pattern of brain atrophy in patients treated with both reconstitution therapies. The results of previous studies mainly assess the degree of whole brain volume loss compared to a comparator in the form of interferon beta 1-a or placebo. In the CAMMS223 study, in which previously untreated patients with RRMS were given interferon beta 1a or alemtuzumab, from 12 to 36 months of follow-up, brain volume increased in the alemtuzumab group and decreased in the interferon beta−1a group (*p* = 0.02) (CAMMS223 Trial Investigators et al., 2008). In the CARE-MS I and II extension study, annual median brain volume loss (BVL) after 5 years of treatment remained low (Coles et al., 2017; Havrdova et al., 2017). Extension studies comparing the efficacy of alemtuzumab with interferon beta 1a also have shown persistently low rates of brain atrophy in the absence of continuous treatment with alemtuzumab or other DMTs during the follow-up period (Traboulsee et al., 2016). In TOPAZ study, the median annual percentage change from the previous year in brain parenchymal fraction (BPF) stabilized over time (Coles et al., 2023). At the first year of follow-up, the median percentage change from baseline in BPF was −2.20% (95% CI −2.51 to −2.06) in the alemtuzumab group and −2.30 (95% CI −2.45 to −1.78) in the interferon-alemtuzumab group. The authors did not comment on the change in the rate of atrophy inhibition by ALEM after the second year of follow-up.

Analysis of the CLARITY trial showed that CLAD treatment reduced whole brain atrophy compared with placebo, and this was associated with a lower risk of disability progression in RRMS (De Stefano et al., 2018). Patients with a lower percentage of brain volume change/year (PBVC/y) were most likely to remain free of disability progression after 2 years and vice versa. A total of 1,326 RRMS patients were included in this study. Volumetric analysis was performed using SIENA software, and the period from 0 to 6 months of follow-up was excluded from the analysis. 1,025 (77.3%) patients were included in the brain volume (BV) analysis (CLAD 3.5 mg/kg, *n* = 336; CLAD tablets 5.25 mg/kg, *n* = 351; placebo, *n* = 338). BVL was significantly reduced in patients treated with CLAD 3.5 mg/kg (−0.77% ± 0.94%, *p* = 0.02, *n* = 336) and 5.25 mg/kg (−0.77 ± 0.95%, *p* = 0.02, *n* = 351) compared to patients treated with placebo (−0.95% ± 1.06%, *n* = 338). When the subgroup of patients with Gd+ lesions at baseline was evaluated, PBVC from 6 to 24 months did not differ between the three groups of patients (CLAD 3.5 mg/kg: −0.92% ± 1.02%, *n* = 110; CLAD 5.25 mg/kg: −1.00% ± 1.08%, *n* = 115; placebo: −0.97% ± 0.97%, *n* = 106). When patients were divided into tertiles of PBVC/year, the tertile with the lowest BVL (PBVC/year > −0.4%) showed the highest rate of patients free from disability progression at 24 months (89%), and the tertile with the highest BVL (PBVC/year < −1.08%) showed the lowest rate of patients free from disability progression (79%). In the ORACLE-MS study, in which 903 patients from 160 MS treatment centres were recruited over a two-year period, percentage brain volume loss (PBVL) was higher in the CLAD 3.5 mg/kg group (−0.48%) than in the placebo group (−0.33%) from 0 to 48 months (Leist et al., 2014). However, PBVL was higher in the placebo group (−0.37%) than in the CLAD group (−0.2%) from 48 to 96 months of follow-up. As mentioned above, in the study by Cortese et al. (2023), during the first 6 months of follow-up, the decrease in percent gray matter volume change (PGMVC) was greater in patients treated with CLAD 3.5 mg/kg than in the placebo group (∆52%, *p* = 0.045), whereas there was no difference in percent white matter volume change (PWMVC) between the two groups (∆31%, *p* = 0.137). A more pronounced difference in GM volume decrease at month 6 between treatment and placebo was found in patients with larger demyelinating lesion volume at baseline than in patients with smaller lesion volume at baseline (*p* = 0.049). Considering the period from 6 to 24 months, brain volume loss was reduced in patients treated with CLAD 3.5 mg/kg compared with those treated with placebo, with a significant difference only in GM. PGMVC for CLAD 3.5 mg/kg was −0.90 ± 0.13% compared with placebo −1.27 ± 0.14% (∆29%, *p* = 0.026), whereas PWMVC for cladribine tablets 3.5 mg/kg was −0.32 ± 0.16% compared with placebo −0.40 ± 0.13% (∆21%, *p* = 0.52). In this study, after a short period of atrophy, GM volume loss progressed at a slower rate in RRMS patients treated with CLAD than in those treated with placebo. In contrast, significant changes were not as evident in the WM compartment. Indeed, many previous studies have shown that changes in brain GM volume are more pronounced and clinically relevant than changes in WM volume and that a pronounced loss of GM volume is associated with long-term disability and seems to better explain physical and cognitive disability than WM and whole-brain atrophy. In the study by Raji and Winkler (2023), 62 patients with RRMS treated with CLAD underwent a 24-month follow-up. Most of them had not been previously treated, the average length of the disease was 6.3 years, the average age was 40 years. In this study, it was observed that the total brain volume began to increase after 12 months of therapy, a statistically significant increase was observed after 24 months of treatment (*p* < 0.006). An increase in WM was noted from the 6th month, statistically significant from the 18th month (*p* < 0.004). GM atrophy was noted after 6 months of treatment, but after 12 and 18 months its volume was equal to the initial one, while after 24 months of follow-up there was no statistically significant change in its volume (*p* < 0.143). Thalamic atrophy was observed after 12 months of treatment, but after 18 months of follow-up, an increase in its volume was observed, which was statistically significant after 24 months of therapy (*p* < 0.019). No changes in the volume of the corpus callosum were observed after both 6 and 12 months of follow-up, but an increase in its volume was observed after 18 months of treatment (*p* < 0.044). Similar to our study, an increase in the volume of the hippocampus was noted in the subsequent months of observation. In contrast, in the study by Filippi et al. (2000), no significant effect of CLAD on changes in brain volume over time was found in patients with progressive MS. It was found that brain volume loss occurs even in a short period of time in progressive MS and CLAD treatment is not able to significantly change this process. It was also suggested that inflammation and the formation of new demyelinating lesions visible in MRI play a marginal role in the development of brain atrophy in patients with progressive MS.

The presented study seems to be innovative. Currently, there is a lack of data in the literature comparing the effect of reconstitution therapies on the degree of brain atrophy in patients with RRMS and assessing the atrophy pattern so precisely. In addition, by excluding from the analysis studies performed after the age of 55, studies of poor quality and maintaining appropriate intervals both between MRI examination and between MRI examination and intravenous administration of glucocorticosteroids, the risk of interference of the presented analysis by external factors was reduced. Moreover, the analysis concerned data collected at equal, annual intervals during a 3-year observation period, which made it possible to monitor the dynamics of volumetric changes and to assess the short-term impact of therapy on the degree and pattern of atrophy. Furthermore, the obtained results are repeatable, which was confirmed by their consistency with our previous observations received using other volumetric software (volBrain) (Pogoda-Wesołowska et al., 2024a,b).

This study had several limitations. One of them was the small number of both groups, especially in the last year of follow-up. Moreover, as mentioned earlier, this was a retrospective real world evidence (RWE) study, burdened with a risk of selection bias. Some of the data from the analysis had to be excluded to control for potential confounding factors that could distort the assessment of the pattern and degree of atrophy in the compared reconstitution therapies. For this reason, during the data analysis MRI examinations performed less than 8 weeks after the administration of intravenous steroids were not taken into account, to reduce the phenomenon of pseudoatrophy. In addition, imaging examinations performed after the age of 55 years were also excluded in order to limit the influence of physiological loss of brain volume. Furthermore, patients previously treated with highly active therapies were not included in the calculations to avoid the influence of this treatment on the loss of brain volume. MRI examinations of low technical quality and those performed at incorrect time intervals were also excluded to minimize the risk of artifacts that could have an influence on the obtained results. Although, all the restrictions caused a decrease in the size of the analyzed group, they increased the reliability of the results. In addition, this was a pilot study and performing analyses, such as correlating brain volume changes results with cognitive function assessments, was not possible at the retrospective stage of the research. To properly perform such correlation, it would be necessary to assess the cognitive function of the patients before the start of the therapy and at subsequent MRI time points, which was currently not possible because the patients were already undergoing treatment. Therefore, further prospective studies with larger groups of patients are needed to fully understand the impact of reconstitution therapies on the neurodegenerative process in patients with RRMS.

In summary, IRT therapy seems to be promising in terms of inhibiting atrophy in patients with RRMS by saving the GM and cortex and by increasing the volume of subcortical structures such as the thalamus and hippocampus. This study also shows that the dynamics of changes in the degree and pattern of atrophy and the effect of DMTs on these phenomena is most intense in the first year of therapy and may be associated with the strong anti-inflammatory activity of the treatment. However, the most visible is the inhibition of the atrophy process by CLAD and ALEM in the subsequent years of treatment, which was confirmed in the above study.

## Data Availability

The data analyzed in this study is subject to the following licenses/restrictions: the dataset is the property of the Military Institute of Medicine – National Research Institute. The results of the presented analysis concern anonymized data. Requests to access these datasets should be directed to apogoda-wesolowska@wim.mil.pl.

## Footnotes

^1^ http://surfer.nmr.mgh.harvard.edu
