# Supplementary material for: Assessment of the impact of reconstitution therapies—cladribine tablets and alemtuzumab—on the atrophy progression among patients with relapse-remitting multiple sclerosis
Source: Front Neurosci. 2025 Feb 27;19:1531163. doi: 10.3389/fnins.2025.1531163 (PMC11903439; doi:10.3389/fnins.2025.1531163)
Supplement: Supplementary file 1 [file Table_1.docx]

***Supplementary Material***

**Table 1.** The results of the group comparison for all analyzed brain structures, along with the corresponding Cohen’s d coefficients and post-hoc power analysis.

|  | Mean (CLAD) | mean (ALEM) | n (CLAD) | n (ALEM) | SD (CLAD) | SD (ALEM) | Cohen’s d | Post-hoc Power |
| --- | --- | --- | --- | --- | --- | --- | --- | --- |
| BrainSeg | -1.13241541 | -1.164403918 | 8 | 7 | 1.97752727 | 1.252828096 | 0.01901473693 | 0.05014435013 |
| TotalGray | -2.191656108 | 0.2590899704 | 8 | 7 | 3.216443928 | 3.113751403 | 0.773237528 | 0.3022862071 |
| Cortex | -2.492306896 | 1.279386939 | 8 | 7 | 3.976109508 | 4.208649558 | 0.9232849862 | 0.4053033858 |
| SubCortGray | -0.8632186858 | -2.461722078 | 8 | 7 | 2.153656665 | 3.414557678 | 0.5694908335 | 0.1858762083 |
| Cerebellar cortex | -1.476776786 | -2.167200226 | 8 | 7 | 4.857183947 | 4.153786091 | 0.1518721685 | 0.05925663856 |
| WM Total | 0.1534398695 | -2.987818994 | 8 | 7 | 6.353525918 | 5.596607969 | 0.5221486125 | 0.1637267891 |
| Cerebral WM | 0.3707210102 | -3.069098205 | 8 | 7 | 6.639142186 | 5.852247316 | 0.5470269581 | 0.1751199783 |
| Cerebellar WM | -3.836187049 | -1.074453941 | 8 | 7 | 4.593461988 | 4.988567528 | 0.5777831316 | 0.1899580154 |
| WM-hypo | -9.370638081 | -21.56572865 | 8 | 7 | 17.01881694 | 22.79597963 | 0.6129799189 | 0.2079408172 |
| Hippocampus | 2.365718782 | -3.572029015 | 8 | 7 | 1.63655323 | 4.886569073 | 1.68193727 | 0.8781648756 |
| Thalamus | 0.2768043131 | 1.192449522 | 8 | 7 | 5.76977735 | 7.868866459 | 0.1342715451 | 0.05722728948 |
| Amygdala | 0.02219569647 | -7.926429819 | 8 | 7 | 4.681860018 | 5.344838159 | 1.590110538 | 0.8403902459 |
| Ventral DC | -2.979654915 | -3.303105249 | 8 | 7 | 2.715663529 | 2.359719936 | 0.1264690727 | 0.056408797 |
| Accumbens area | -6.264099325 | 5.376271841 | 8 | 7 | 9.637960486 | 14.60213409 | 0.9554516116 | 0.428589853 |
| Pallidium | 0.3916621003 | -4.769824613 | 8 | 7 | 12.52127108 | 14.05830177 | 0.3894627143 | 0.1123426993 |
| Putamen | -0.7387437111 | 0.1720517004 | 8 | 7 | 6.65499221 | 8.82871579 | 0.1177569425 | 0.05555355291 |
| Caudate | -2.896964479 | -0.9365378255 | 8 | 7 | 6.248577089 | 11.16736248 | 0.2211503285 | 0.0697368929 |
| CC | 8.215582855 | 5.516735568 | 8 | 7 | 28.02721445 | 29.86715056 | 0.09341487545 | 0.05349074107 |
| Lateral ventricle | 12.64349507 | 3.587579387 | 8 | 7 | 17.57626148 | 24.7806219 | 0.427011285 | 0.1252733011 |
| VentricleChoroidVol | 11.10640267 | 3.682276241 | 8 | 7 | 16.66182363 | 25.26269013 | 0.3523171009 | 0.1007962002 |
| CSF | -2.491505502 | -11.27147039 | 8 | 7 | 10.77163461 | 15.82809493 | 0.6578917066 | 0.2323922404 |

BrainSeg - total brain volume, TotalGray- gray matter total volume, SubCortGray - subcortical gray matter volume, WM total - total volume of white matter, Cerebral WM - volume of white matter of cerebrum, Cerebellar WM - volume of white matter of cerebellum, WM-hypo+hypointense WM lesions, Ventral DC – ventral diencephalon, CC – corpus callosum, CSF – cerebrospinal fluid.
